# Comparison of crowd-sourced, electronic health records based, and traditional health-care based influenza-tracking systems at multiple spatial resolutions in the United States of America

**DOI:** 10.1186/s12879-018-3322-3

**Published:** 2018-08-15

**Authors:** Kristin Baltrusaitis, John S. Brownstein, Samuel V. Scarpino, Eric Bakota, Adam W. Crawley, Giuseppe Conidi, Julia Gunn, Josh Gray, Anna Zink, Mauricio Santillana

**Affiliations:** 1 Computational Health Informatics Program, Boston Children’s Hospital, Boston, MA 02115 USA; 2 Department of Biostatistics, Boston University School of Public Health, 801 Massachusetts Avenue 3rd Floor, Boston, MA 02118 USA; 3 Harvard Medical School, Boston, MA 02115 USA; 4 Department of Mathematics and Statistics, University of Vermont, Vermont, USA; 5 City of Houston Health Department, Houston, TX 77054 USA; 6 Skoll Global Threats Fund, San Francisco, CA USA; 7 Boston Public Health Commission, Boston, MA USA; 8 athenaResearch at athenahealth, Watertown, MA USA

**Keywords:** Community-based participatory research, Crowd-sourcing, Digital disease detection, Influenza, Public health informatics, Public health surveillance

## Abstract

**Background:**

Influenza causes an estimated 3000 to 50,000 deaths per year in the United States of America (US). Timely and representative data can help local, state, and national public health officials monitor and respond to outbreaks of seasonal influenza. Data from cloud-based electronic health records (EHR) and crowd-sourced influenza surveillance systems have the potential to provide complementary, near real-time estimates of influenza activity. The objectives of this paper are to compare two novel influenza-tracking systems with three traditional healthcare-based influenza surveillance systems at four spatial resolutions: national, regional, state, and city, and to determine the minimum number of participants in these systems required to produce influenza activity estimates that resemble the historical trends recorded by traditional surveillance systems.

**Methods:**

We compared influenza activity estimates from five influenza surveillance systems: 1) patient visits for influenza-like illness (ILI) from the US Outpatient ILI Surveillance Network (ILINet), 2) virologic data from World Health Organization (WHO) Collaborating and National Respiratory and Enteric Virus Surveillance System (NREVSS) Laboratories, 3) Emergency Department (ED) syndromic surveillance from Boston, Massachusetts, 4) patient visits for ILI from EHR, and 5) reports of ILI from the crowd-sourced system, Flu Near You (FNY), by calculating correlations between these systems across four influenza seasons, 2012–16, at four different spatial resolutions in the US. For the crowd-sourced system, we also used a bootstrapping statistical approach to estimate the minimum number of reports necessary to produce a meaningful signal at a given spatial resolution.

**Results:**

In general, as the spatial resolution increased, correlation values between all influenza surveillance systems decreased. Influenza-like Illness rates in geographic areas with more than 250 crowd-sourced participants or with more than 20,000 visit counts for EHR tracked government-lead estimates of influenza activity.

**Conclusions:**

With a sufficient number of reports, data from novel influenza surveillance systems can complement traditional healthcare-based systems at multiple spatial resolutions.

**Electronic supplementary material:**

The online version of this article (10.1186/s12879-018-3322-3) contains supplementary material, which is available to authorized users.

## Background

Every year influenza epidemics are responsible for substantial clinical and economic burdens in the United States of America (US) [1]. Consequently, local, state, and national health authorities require quantitative evidence that is timely and representative to make informed decisions regarding the selection and allocation of resources. The Centers for Disease Control and Prevention (CDC), a governmental agency, has been continuously collecting information on the number of outpatient visits for influenza-like illness (ILI) from a diverse network of healthcare providers as well as on the number of influenza-positive lab specimens from public health and clinical laboratories across the US for multiple decades [2]. Although influenza surveillance occurs throughout the calendar year, the influenza season is defined by the Morbidity and Mortality Weekly Report (MMWR) week 40 through week 20, which corresponds with months October through May. Due to the time to collect, process, and aggregate this information, CDC influenza surveillance reports are traditionally published with a 1–2 week delay. Alternative data sources that are available in near-real time may aid in the design, initiation, or communication of timely strategies and mitigate the impact of influenza.

Over the past decade, Internet-based technologies have been explored as new ways to monitor influenza activity and provide more immediate estimates of disease activity. These Internet-based technologies include systems such as Yahoo [3], Google [4–6], Baidu [7], Twitter posts [8–10], clinicians’ database queries [11], cloud-based Electronic Health Records (EHR) [12], and online participatory cohorts that allow individuals to report symptoms [13–15]. The ability of these novel Internet-based and crowd-sourced approaches to complement, track, and forecast traditional provider-based influenza surveillance systems has been established at the national and regional levels in the US [12, 15–20]. However, because characteristics of activity may differ across states and sub-populations [21], further investigation of these novel systems is essential at finer spatial resolutions [22].

In this paper, we evaluate two novel influenza-tracking systems, athenahealth, a cloud-based EHR-based system, and Flu Near You (FNY), a crowd-sourced system. Founded in 1997, athenahealth is a provider of cloud-based services and mobile applications for medical groups and health systems. Similar to traditional health-care based surveillance systems, athenahealth collects data on individuals who seek medical care. Because athenahealth’s network is cloud-based, the proportion of patients with ILI symptoms in their national network of providers can be estimated in near real-time, potentially providing estimates of influenza activity faster than the national surveillance systems (https://insight.athenahealth.com/flu-dashboard-2016). Flu Near You is an online crowd-sourced surveillance system that allows volunteers in the US and Canada to report weekly if they have experienced ILI symptoms [15]. The majority (65%) of FNY respondents who report ILI do not seek medical attention, therefore, this system captures illness activity among a population not routinely included among the other healthcare-based systems considered in this paper.

The objectives of this paper are to assess whether these novel systems, EHR and crowd-sourced, correlate with traditional influenza surveillance systems across multiple spatial resolutions with different sample sizes and to determine the minimum number of visits or reports necessary in each of these novel systems to produce influenza activity estimates that resemble the historical trends recorded by traditional surveillance systems for a given spatial resolution.

## Methods

### Data

#### Electronic health records (EHR) from athenahealth

Weekly state-aggregated counts of total visits, influenza vaccine visits, influenza visits, ILI visits, and unspecified viral or ILI visits were provided by the athenahealth research team for the time period of 2012–16 (http://www.athenahealth.com). For the analysis presented in this paper, ILI was defined as *Unspecified Viral or ILI Visit Count*, which included the number of visits where the patient had an unspecified viral diagnosis, an influenza diagnosis, or a fever diagnosis with an accompanying sore throat or cough diagnosis. Influenza-Like Illness rates for a given location were calculated by dividing the *Unspecified Viral or ILI Visit Count* by the total number of visits.

#### Crowd-sourced from flu near you

Flu Near You was created in 2011 through collaboration between HealthMap of Boston Children’s Hospital and the Skoll Global Threats Fund (https://flunearyou.org/). [15] This system maintains a website and mobile application that allows volunteers in the United States and Canada to report the health information of the user and their family using a brief weekly survey. Flu Near You ILI rates were calculated by dividing the number of participants reporting ILI, defined by a symptom report of fever plus cough and/or sore throat, in a given week, by the total number of FNY participant reports in that same week at each spatial resolution. Participants were aggregated at each spatial resolution using the zip code provided at registration for the time-period of 2012–16.

#### CDC ILINet national/regional/state

Information on patient visits to health care providers for ILI is collected through the US Outpatient Influenza-like Illness Surveillance Network (ILINet, https://www.cdc.gov/flu/weekly/overview.htm) [2]. For this system, ILI was defined as fever (temperature of 37.8 °C [100 °F] or greater) and a cough and/or a sore throat without a known cause other than influenza. Weighted percent ILI, calculated by weighting the percentage of patient visits to healthcare providers for ILI reported each week on the basis of state population, was used as the influenza activity measure. For regional analyses, we used the ten Health and Human Services (HHS) defined regions. Each region consists of three to eight US states or territories.

#### CDC virology

Virological influenza surveillance data is collected through participating US World Health Organization (WHO) Collaborating Laboratories and National Respiratory and Enteric Virus Surveillance System (NREVSS) laboratories located throughout the US. The number of specimens testing positive for influenza was used in these analyses [2].

#### Boston epidemiological data

The Boston Public Health Commission (BPHC) has operated a syndromic surveillance system since 2004. All nine acute care Boston hospitals electronically send limited data for all emergency department (ED) visits every 24 h. Data sent includes visit date, chief complaint, zip code of residence, age, gender, and race/ethnicity. Influenza-Like Illness visits were defined as fever and a cough or sore throat using chief complaints. Greater Boston was defined as zip codes associated with Suffolk, Norfolk, Middlesex, Essex, and Plymouth counties. These zip codes are associated with over 90% of Boston ED visits. Influenza-Like Illness rates for Greater Boston were calculated by using the number of ILI visits divided by the total number of ED visits.

### Statistical analysis

#### Correlation with traditional influenza surveillance systems across multiple spatial resolutions with different sample sizes

We used Pearson correlations to compare EHR and crowd-sourced ILI rates to ILI rates from ILINet along with the number of specimens testing positive for influenza from the virologic surveillance system. Correlations were calculated at the national and HHS-defined regional resolutions during the time period of October 1, 2012 through May 21, 2016, and for each of the four individual influenza seasons within this time period (MMWR weeks 40 to 20) separately. We also present comparisons of EHR to CDC ILINet for 46 states and comparisons of crowd-sourced ILI to CDC ILINet for 49 states that voluntarily provided historic data across all seasons. Finally, crowd-sourced ILI rates were also compared to ILI rates estimated from ED visits in the Greater Boston area. Boston was chosen as a pilot city because of the large FNY user base and availability of data. Electronic Health Record data at this spatial resolution was not available.

Spatial resolutions were classified into three author-defined categories based on correlation values with CDC ILINet across all seasons. Spatial resolutions with correlations less than 0.5 were classified as “poor”, spatial resolutions with correlations between 0.5 and 0.70 were classified as “good”, and spatial resolutions with correlations greater than or equal to 0.70 were classified as “excellent”. Data were analyzed using R, version 3.3.2, [23] and descriptive statistics are presented as median (Interquartile Range, IQR).

#### Bootstrapping approach to estimate the minimum number of crowd-sourced reports necessary to produce estimates that resemble the historical government-lead surveillance system trends

As above, weekly ILI rates from the crowd-sourced system were compared to weighted ILI rates from CDC ILINet and the number of specimens testing positive for influenza at national and regional resolutions during the 2015–16 influenza season. State and city resolutions were not included in this analysis because the crowd-sourced user base was not large enough. At the national level, Pearson correlations were calculated for subsets of the crowd-sourced data from 0.1 to 15% of the full dataset in increments of 0.1%, and at the regional level, Pearson correlations were calculated from 1 to 100% of the full dataset in increments of 1%. This process was repeated 1000 times using sampling with replacement (bootstrapping), stratified by week at each spatial resolution. The 95% confidence intervals were calculated by ordering the Pearson correlation coefficients and selecting the 2.5th and 97.5th percentiles. This method was not performed for EHR because available data was aggregated at the state level.

## Results

### Correlation with traditional influenza surveillance systems across multiple spatial resolutions with different sample sizes

#### Electronic health records

Pearson correlations between CDC ILINet and EHR and mean weekly visits at all spatial resolutions are shown in Additional file 1: Table S1. Across all seasons, the national mean weekly visits was 863,361, and the national correlation was 0.97. At the regional level, the median of the mean weekly visits was 69,077 (IQR: 26,584, 126,455). Region 7 (Iowa, Kansas, Missouri, and Nebraska) had the smallest mean weekly visits (10,177) and Region 4 (Alabama, Florida, Georgia, Kentucky, Mississippi, North Carolina, South Carolina, and Tennessee) had the largest mean weekly visits (195,142). The median regional correlation was 0.93 (IQR: 0.91, 0.95), and all regions were classified as “excellent”. At the state level, the median of the mean weekly visits was 11,840 (IQR: 4204, 30,740), and the median correlation was 0.86 (IQR: 0.80, 0.92). Using the cutoff values defined in the methods sections, 41 of the states with data available were classified as “excellent” and five were classified as “good”.

#### Crowd-sourced reports

Pearson correlations of crowd-sourced ILI rates versus CDC ILINet and BPHC as well as mean weekly reports at all spatial resolutions are shown in Additional file 1: Table S2. The national mean weekly reports across all seasons was 9699, and the correlation was 0.81. At the regional level, the median of the mean weekly reports was 889 (IQR: 707, 1157). Region 7 had the smallest mean number of weekly reports (415), and Region 4 had the largest mean number of weekly reports (1798). The median correlation was 0.74 (IQR: 0.73, 0.76). Across all seasons, 9 regions were classified as “excellent” and one region was classified as “good”. The median of the mean weekly reports at the state level was 128 (IQR: 57, 263), and the median correlation with CDC ILINet was 0.55 (IQR: 0.43, 0.63). Two states, Massachusetts and California, were classified as “excellent”, 26 states were classified as “good”, and 21 states were classified as “poor”.

**Fig. 1:**
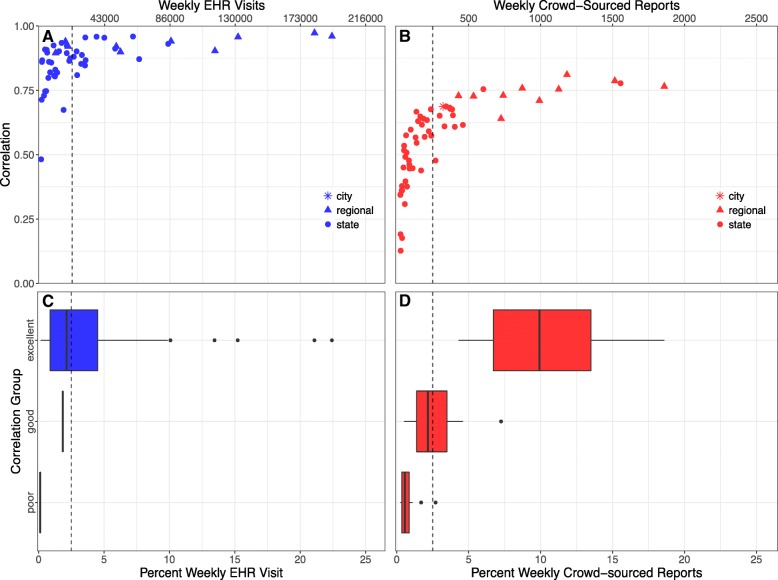
**a** Correlation of EHR (blue) with CDC ILINet by mean visits at the regional and state resolution across all seasons (**b**) Correlation of the crowd-sourced system (red) with CDC ILINet and BPHC (star) by mean reports at the regional, state, and city resolutions across all seasons (**c**) EHR correlation category (blue) by percent of total visits (**d**) Crowd-sourced correlation category (red) by percent of total reports

Fig. 1a and b display correlations across all seasons plotted as a function of the mean weekly visits (EHR) or mean weekly reports (FNY). As shown in this figure, in general, correlation values increased as the mean weekly visits or reports increased for both EHR and crowd-sourced at all the regional and state resolutions across all seasons. For EHR, spatial resolutions with at least 2.5% (approximately 20,000/863,361) of total weekly visits are more likely to be classified as “excellent” compared to “good” or “poor” (Fig. 1c). Spatial resolutions with at least 2.5% (approximately 250/ 9699) of total weekly crowd-sourced reports are more likely to be classified as “good” compared to “poor”, and spatial resolutions with at least 5% (approximately 500/9699) of weekly crowd-sourced reports are more likely to be classified as “excellent” compared to “good” or “poor” (Fig. 1d).

**Fig. 2:**
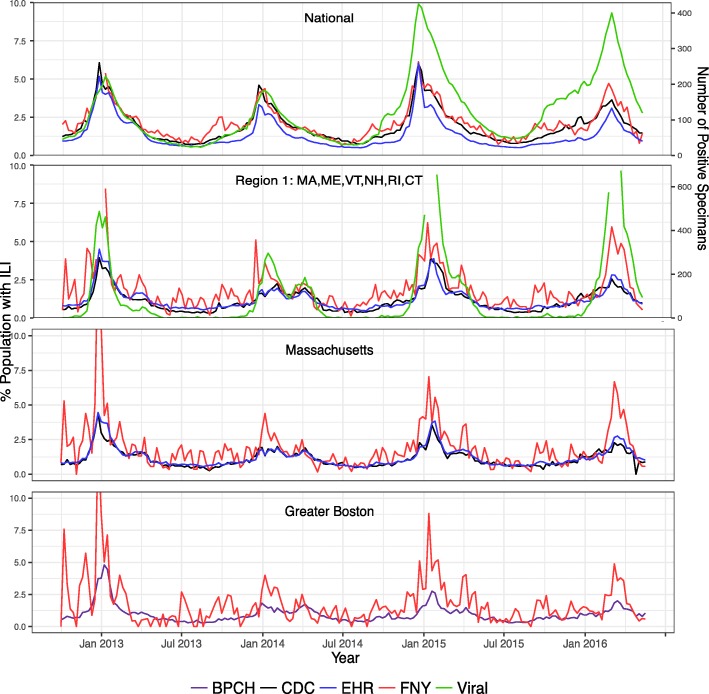
Percent ILI for CDC ILINet (black), EHR (blue), crowd-sourced FNY (red), and BPHC (purple) as well as number of viral specimens (green) as a function of time for National, Region 1, Massachusetts, and Greater Boston spatial resolutions

Figure 2 provides the time series of CDC ILINet, BPHC, EHR, and crowd-sourced (FNY) ILI rates and CDC number viral specimens across all seasons at four spatial resolutions: National, Region 1 (Connecticut, Maine, Massachusetts, New Hampshire, Rhode Island, and Vermont), Massachusetts, and Greater Boston. Although the amount of noise increases as the spatial resolution increases and the mean weekly visits or reports decrease, a meaningful signal is retained for both EHR and crowd-sourced ILI rates.

### Bootstrapping approach to estimate the minimum number of crowd-sourced reports necessary to produce estimates that resemble the historical government-lead surveillance system trends

**Fig. 3:**
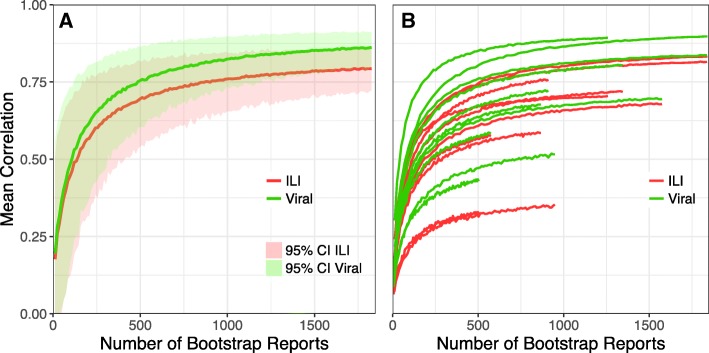
Correlation between bootstrapped samples of crowd-sourced (FNY) estimated percent ILI and observed percent ILI, as reported by the CDC ILINet (red) and the number of viral-positive specimens (green) at the national resolution (**a**) with 95% Confidence Intervals (CI) and the regional resolution (**b**) for the 2015–16 influenza season

During the 2015–16 influenza season, a total of 401,993 crowd-sourced reports were collected in the US, corresponding to a weekly average of 12,182 reports. The Pearson correlation coefficients during the 2015–16 influenza seasons for the full crowd-sourced dataset and CDC ILINet and the number of viral-positive specimens were 0.84 and 0.92, respectively. Figure 3a shows the mean Pearson correlation coefficient and 95% CI of 1000 bootstrap runs between the crowd-sourced system and both CDC ILINet and the number of viral-positive specimens for increasing weekly reports. As shown in this figure, the correlation coefficient increases as the number of weekly reports increases, but the rate of growth slows around 250 weekly reports. Although the crowd-sourced data appear to correlate more strongly with virological data during this influenza season, this pattern is not consistent across all seasons and regions (Additional file 1: Table S3). The correlations for 1800 weekly crowd-sourced reports (approximately 10%) were 0.80 and 0.86 for CDC ILINet and CDC number of viral-positive specimens, respectively. At an arbitrary cut-off value of 250 weekly reports, the correlation between the crowd-sourced system and CDC ILINet was approximately 0.60, and the correlation between the crowd-sourced system and the number of viral-positive specimens was 0.65. When the number of reports is less than this value, correlation coefficients drop-off sharply. A similar pattern is shown at the regional level (Fig. 3b). Although some regions reach saturation at higher correlations than other regions, there is non-linear growth in the correlation coefficient until estimates include approximately 250 weekly reports.

## Discussion

Traditional surveillance systems currently used by governmental agencies are robust, well accepted, and provide the best basis for tracking influenza activity. However, because estimates only include individuals who visit a medical care facility and there is typically a delay from onset of patient symptoms to final publication of reports, alternative data sources have the potential to minimize these delays in reporting and complement these traditional systems. Although there is still a time delay from onset of patient symptoms to presentation at a health-care provider, the EHR cloud-based system allows symptom reports to be aggregated in near-real time. On the other hand, the crowd-sourced system does not include the same time delay as health-care based systems and captures individuals who do not seek medical care. However, while participants have the option to report symptoms the same day as onset, most participants do not report until they receive the weekly reminder and data is typically aggregated once a week.

For both EHR and crowd-sourced ILI, as the number of total reports increases, the correlations with traditional ILI estimates from governmental agencies also increase. However, EHR data showed higher correlations with CDC ILINet and the number of viral-positive specimens compared to crowd-sourced data at similar spatial resolutions. EHR correlations with CDC ILINet are close to one, which shows that healthcare-based influenza surveillance with different data capture strategies lead to similar ILI incidence curves. Although both EHR and the CDC use data from patients seeking medical attention, the proportion of visit settings differs slightly between the two systems, with emergency department visits being under-represented in the EHR. On the other hand, crowd-sourced correlations with CDC ILINet never reach a correlation of one. Instead, crowd-sourced correlations converge to approximately 0.8–0.9, as shown using both empirical and theoretical approaches. A similar observation was observed when comparing methods of provider recruitment in Texas [24]. This difference in correlation saturation may be a result of differences in the activity being measured (e.g. ILI reports out of all persons enrolled vs. visits with ILI out of the total number of patient visits) and the population under surveillance, as the crowd-sourced population includes individuals who may not seek medical attention. Based on preliminary analyses, we estimate that approximately 65% of the FNY population who reported ILI symptoms did not seek medical attention. The Italian crowd-sourced counterpart, INFLUWEB, has also reported that approximately two thirds of their participants did not seek medical assistance [25]. Furthermore, studies in the US have shown that approximately 40% of individuals with ILI seek healthcare [26]. The crowd-sourced population also differs by demographics. Females and middle-aged individuals are over-represented in the crowd-sourced population [27]. In addition, crowd-sourced estimates can be affected by media attention and by user participation. For example, the large peak observed in January 2013 occurred after FNY was featured in NBC’s Nightly News with Brian Williams. Investigators have applied a few methods to adjust for these reporting biases, including dropping first reports and a spike-detector method [15]. We did not adjust for these biases in this paper.

In general, both crowd-sourced and EHR ILI rates showed higher correlations with CDC ILINet compared to the number of viral-positive specimens at the national and regional resolutions (Additional file 1: Table S3). One interesting pattern to note is that when using the bootstrap resampling approach, crowd-sourced correlations with CDC laboratory confirmed influenza specimens reaches the saturation faster than correlations with CDC ILINet. This pattern is also evident at the regional resolution.

Based on the results from this study, we estimate that ILI rates from EHR and crowd-sourced data track traditional ILI estimates from governmental agencies at spatial resolutions that have at least 20,000 weekly EHR visits and 250 weekly crowd-sourced reports. Some spatial resolutions are not well represented in the included novel systems. During the 2015–16 influenza season, for example, 47 states were represented in this EHR network and 26 of these states reached the 20,000 threshold. Although all 50 states are represented in the crowd-sourced system, 32 states did not reach the 250 weekly report threshold during the 2015–16 influenza season. In addition, the geographic distribution of crowd-sourced reports shows large gaps of information especially in the middle and southern areas of the US, and participants tend to cluster around large urban areas, with especially large user bases in the greater metropolitan areas surrounding Boston, New York City, and San Francisco. Flu Near You has made recent efforts to recruit new users through online media campaigns through Facebook, and other previously successful recruitment strategies, such as encouraging current users to recruit friends and colleagues to join, [28] can be easily employed.

Ideally, we would want to compare ILI rates from crowd-sourced reports to laboratory confirmed influenza cases in the general population. Currently, the CDC provides yearly estimates of seasonal influenza burden in the general population using laboratory-confirmed influenza-associated hospitalization rates from their Influenza Hospital Surveillance Network (FluSurv-NET). However, they do not provide weekly estimates to the public of laboratory-confirmed influenza burden. Although the mechanisms of capture differ between the syndromic systems, the general seasonal trends are similar and provide valuable information about changes in influenza activity.

## Conclusions

Our findings suggest that both EHR and crowd-sourced ILI estimates correlate with ILI estimates from traditional influenza surveillance systems in various spatial resolutions with a sufficient number of visits or reports. Spatial resolutions with at least 250 mean weekly crowd-sourced reports display correlations higher than 0.5 with traditional influenza surveillance systems. Furthermore, spatial resolutions with approximately 20,000 weekly EHR visit counts consistently show correlations greater than 0.7 with traditional influenza surveillance systems. As the FNY user base and availability of EHR data are increased throughout the US, these internet-based surveillance tools may become a complementary way to timely monitor influenza activity, especially in populations who do not access health care systems, areas with limited surveillance data, and community based populations.

## Additional file

Additional file 1 **Table S1.** Pearson correlations between EHR and CDC ILINet and average weekly EHR visits at the national, regional, and state resolutions. **Table S2.** Pearson correlations between Crowd-sourced and CDC ILINet/BPHC and average weekly crowd-sourced reports at the national, regional, state, and state resolutions. **Table S3.** Pearson correlations between CDC number positive viral reports and EHR, crowd-sourced (FNY), and CDC ILINet. (DOCX 81 kb)

## Abbreviations

BPHC: Boston Public Health Commission
CDC: Centers for disease control and prevention
ED: Emergency department
EHR: Electronic health records
FluSurv-NET: Influenza Hospital Surveillance Network
FNY: Flu near you
HHS: Health and human services
ILI: Influenza-like illness
ILINet: US outpatient influenza-like illness surveillance network
IQR: Interquartile range
MMWR: Morbidity and mortality weekly report
NREVSS: National respiratory and enteric virus surveillance system
US: United States of America
WHO: World Health Organization
