# Supplementary material for: Comparison of crowd-sourced, electronic health records based, and traditional health-care based influenza-tracking systems at multiple spatial resolutions in the United States of America
Source: BMC Infect Dis. 2018 Aug 15;18:403. doi: 10.1186/s12879-018-3322-3 (PMC6094455; doi:10.1186/s12879-018-3322-3)
Supplement: Supplementary file 1 — Table S1. Pearson correlations between EHR and CDC ILINet and average weekly EHR visits at the national, regional, and state resolutions. Table S2. Pearson correlations between Crowd-sourced and CDC ILINet/BPHC and average weekly crowd-sourced reports at the national, regional, state, and state resolutions. Table S3. Pearson correlations between CDC number positive viral reports and EHR, crowd-sourced (FNY), and CDC ILINet. (DOCX 81 kb) [file 12879_2018_3322_MOESM1_ESM.docx]

**Table S1.** Pearson correlations between EHR and CDC ILINet and average weekly EHR visits at the national, regional, and state resolutions.

| Geographical Resolution | All Seasons | | 2012-13 | | 2013-14 | | 2014-15 | | 2015-16 | |
| --- | --- | --- | --- | --- | --- | --- | --- | --- | --- | --- |
|  | Corr | $\bar{n}$ | Corr | $\bar{n}$ | Corr | $\bar{n}$ | Corr | $\bar{n}$ | Corr | $\bar{n}$ |
| National | 0.97 | 944306 | 0.99 | 626424 | 0.99 | 779460 | 0.99 | 959790 | 0.9 | 1141282 |
| Region 1 | 0.94 | 89520 | 0.99 | 69528 | 0.95 | 85774 | 0.94 | 90053 | 0.9 | 95743 |
| Region 2 | 0.92 | 56454 | 0.97 | 35129 | 0.95 | 44667 | 0.95 | 56086 | 0.9 | 71345 |
| Region 3 | 0.96 | 138514 | 0.98 | 103514 | 0.96 | 124288 | 0.99 | 136097 | 0.77 | 161590 |
| Region 4 | 0.98 | 215644 | 0.97 | 134638 | 0.99 | 166501 | 0.99 | 224983 | 0.96 | 269254 |
| Region 5 | 0.97 | 204504 | 0.99 | 124820 | 0.97 | 160290 | 0.98 | 202922 | 0.97 | 261822 |
| Region 6 | 0.92 | 131956 | 0.98 | 78908 | 0.96 | 103414 | 0.92 | 141293 | 0.42 | 154954 |
| Region 7 | 0.95 | 9668 | 0.96 | 11863 | 0.9 | 7845 | 0.98 | 10643 | 0.83 | 11027 |
| Region 8 | 0.91 | 19059 | 0.97 | 11358 | 0.96 | 16440 | 0.88 | 18256 | 0.94 | 24013 |
| Region 9 | 0.94 | 57122 | 0.93 | 38771 | 0.94 | 51567 | 0.98 | 57691 | 0.97 | 65520 |
| Region 10 | 0.95 | 19131 | 0.81 | 16848 | 0.98 | 17596 | 0.96 | 19754 | 0.89 | 20717 |
| Alaska | *-* | - | *-* | - | *-* | - | *-* | - | *-* | - |
| Alabama | 0.84 | 14309 | 0.98 | 6847 | 0.93 | 7804 | 0.92 | 15829 | 0.92 | 20900 |
| Arkansas | 0.9 | 11836 | 0.81 | 7314 | 0.92 | 8653 | 0.94 | 13151 | 0.84 | 14673 |
| Arizona | 0.88 | 17461 | 0.97 | 17138 | 0.95 | 16599 | 0.96 | 18537 | 0.94 | 18919 |
| California | 0.93 | 33580 | 0.86 | 19078 | 0.93 | 29645 | 0.97 | 32862 | 0.95 | 39854 |
| Colorado | 0.81 | 6941 | 0.94 | 4454 | 0.94 | 5551 | 0.69 | 6644 | 0.87 | 9300 |
| Connecticut | 0.77 | 11592 | 0.9 | 5477 | 0.6 | 10220 | 0.72 | 10347 | 0.68 | 14259 |
| Delaware | 0.7 | 3549 | 0.81 | 1369 | 0.81 | 2906 | 0.73 | 3619 | 0.92 | 4095 |
| Florida | *-* | 69367 | *-* | 36132 | *-* | 52630 | *-* | 73416 | *-* | 90055 |
| Georgia | 0.9 | 31259 | 0.92 | 21018 | 0.97 | 22224 | 0.92 | 31852 | 0.77 | 42019 |
| Hawaii | 0.66 | 2341 | 0.08 | 1363 | 0.87 | 1538 | 0.94 | 2550 | 0.86 | 2860 |
| Iowa | 0.55 | 1153 | 0.83 | 685 | 0.8 | 994 | 0.43 | 1552 | 0.63 | 1111 |
| Idaho | 0.64 | 258 | *-* | 37 | *-* | 27 | *-* | 103 | 0.64 | 763 |
| Illinois | 0.95 | 38450 | 0.98 | 31132 | 0.93 | 35840 | 0.99 | 39224 | 0.94 | 42160 |
| Indiana | 0.95 | 38088 | 0.97 | 13788 | 0.94 | 29224 | 0.98 | 40031 | 0.88 | 43916 |
| Kansas | 0.9 | 2126 | 0.96 | 6361 | 0.91 | 1807 | 0.91 | 1893 | 0.64 | 2871 |
| Kentucky | 0.83 | 7023 | 0.93 | 13133 | 0.95 | 9523 | 0.91 | 6299 | 0.89 | 6877 |
| Louisiana | 0.92 | 8966 | 0.95 | 8708 | 0.94 | 9829 | 0.9 | 9070 | 0.78 | 9241 |
| Massachusetts | 0.96 | 60657 | 0.96 | 56545 | 0.96 | 60461 | 0.97 | 61900 | 0.94 | 62223 |
| Maryland | 0.74 | 17932 | 0.63 | 11225 | 0.81 | 14144 | 0.93 | 16498 | 0.36 | 24346 |
| Maine | 0.86 | 8609 | 0.9 | 1706 | 0.89 | 7707 | 0.92 | 8667 | 0.52 | 9577 |
| Michigan | 0.86 | 35790 | 0.94 | 6916 | 0.89 | 10149 | 0.95 | 35331 | 0.97 | 66280 |
| Minnesota | *-* | - | *-* | - | *-* | - | *-* | - | *-* | - |
| Missouri | 0.92 | 4939 | 0.95 | 4152 | 0.86 | 3928 | 0.98 | 5607 | 0.72 | 5354 |
| Mississippi | 0.92 | 18009 | 0.94 | 7098 | 0.94 | 10348 | 0.98 | 21003 | 0.83 | 22400 |
| Montana | *-* | - | *-* | - | *-* | - | *-* | - | *-* | - |
| North Carolina | 0.92 | 29628 | 0.86 | 18206 | 0.94 | 28360 | 0.97 | 27928 | 0.87 | 33888 |
| North Dakota | 0.65 | 3918 | 0.68 | 1877 | 0.86 | 3635 | 0.57 | 3978 | 0.68 | 4356 |
| Nebraska | 0.8 | 1450 | 0.60 | 665 | 0.7 | 1117 | 0.84 | 1591 | 0.6 | 1691 |
| New Hampshire | 0.83 | 2029 | 0.86 | 335 | 0.59 | 1524 | 0.89 | 2107 | 0.86 | 2418 |
| New Jersey | 0.87 | 34190 | 0.94 | 21531 | 0.87 | 25719 | 0.92 | 35903 | 0.93 | 42952 |
| New Mexico | 0.85 | 8821 | 0.87 | 5728 | 0.9 | 6130 | 0.89 | 9603 | 0.9 | 11202 |
| Nevada | 0.8 | 3740 | 0.74 | 2266 | 0.69 | 3785 | 0.93 | 3743 | 0.9 | 3887 |
| New York | 0.86 | 22264 | 0.93 | 13598 | 0.89 | 18948 | 0.93 | 20183 | 0.82 | 28393 |
| Ohio | 0.93 | 85635 | 0.94 | 72593 | 0.96 | 84633 | 0.92 | 85081 | 0.95 | 91085 |
| Oklahoma | 0.87 | 30213 | 0.93 | 5817 | 0.93 | 23646 | 0.81 | 34687 | 0.67 | 28762 |
| Oregon | 0.81 | 5741 | 0.79 | 5380 | 0.93 | 5231 | 0.84 | 6325 | 0.66 | 5520 |
| Pennsylvania | 0.9 | 48344 | 0.96 | 46352 | 0.9 | 48756 | 0.94 | 48271 | 0.79 | 48090 |
| Rhode Island | 0.92 | 5210 | 0.92 | 4229 | 0.84 | 4774 | 0.94 | 5484 | 0.93 | 5648 |
| South Carolina | 0.94 | 18586 | 0.96 | 14630 | 0.94 | 17338 | 0.98 | 18106 | 0.78 | 21859 |
| South Dakota | 0.79 | 4490 | 0.55 | 3705 | 0.66 | 4336 | 0.94 | 4339 | 0.63 | 4736 |
| Tennessee | 0.91 | 27463 | 0.92 | 17575 | 0.96 | 18275 | 0.92 | 30551 | 0.81 | 31257 |
| Texas | 0.86 | 72121 | 0.98 | 51341 | 0.96 | 55156 | 0.85 | 74783 | 0.14 | 91076 |
| Utah | 0.81 | 1956 | 0.94 | 1374 | 0.85 | 1386 | 0.93 | 1472 | 0.78 | 3715 |
| Virginia | 0.98 | 47939 | 0.95 | 29662 | 0.98 | 38007 | 0.98 | 46367 | 0.9 | 63646 |
| Vermont | 0.75 | 1423 | 0.8 | 1237 | 0.8 | 1089 | 0.86 | 1547 | 0.71 | 1618 |
| Washington | 0.92 | 13132 | 0.82 | 11431 | 0.92 | 12338 | 0.94 | 13326 | 0.77 | 14434 |
| Wisconsin | 0.64 | 6541 | *-* | 392 | 0.35 | 444 | 0.55 | 3256 | 0.86 | 18381 |
| West Virginia | 0.95 | 20750 | 0.92 | 14905 | 0.96 | 20475 | 0.98 | 21343 | 0.89 | 21412 |
| Wyoming | 0.77 | 1754 | 0.91 | 1143 | 0.84 | 1533 | 0.74 | 1823 | 0.62 | 1906 |

HHS regions: Region 1 Connecticut, Maine, Massachusetts, New Hampshire, Rhode Island, Vermont; Region 2: New Jersey, New York, Puerto Rico, US Virgin Islands; Region 3: Delaware, District of Columbia, Maryland, Pennsylvania, Virginia, West Virginia; Region 4: Alabama, Florida, Georgia, Kentucky, Mississippi, North Carolina, South Carolina, Tennessee; Region 5: Illinois, Indiana, Michigan, Minnesota, Ohio, Wisconsin; Region 6: Arkansas, Louisiana, New Mexico, Oklahoma, Texas; Region 7: Iowa, Kansas, Missouri, Nebraska; Region 8: Colorado, Montana, North Dakota, South Dakota, Utah, Wyoming; Region 9: Arizona, California, Guam, Hawaii, Nevada; and Region 10: Alaska, Idaho, Oregon, Washington

**Table S2.** Pearson correlations between Crowd-sourced and CDC ILINet/ BPHC and average weekly crowd-sourced reports at the national, regional, state, and state resolutions.

| Geographical Resolution | All Seasons | | 2012-13 | | 2013-14 | | 2014-15 | | 2015-16 | |
| --- | --- | --- | --- | --- | --- | --- | --- | --- | --- | --- |
|  | Corr | $\bar{n}$ | Corr | $\bar{n}$ | Corr | $\bar{n}$ | Corr | $\bar{n}$ | Corr | $\bar{n}$ |
| National | 0.81 | 9699 | 0.78 | 9987 | 0.91 | 10291 | 0.97 | 10676 | 0.84 | 12182 |
| Region 1 | 0.71 | 958 | 0.77 | 775 | 0.34 | 1047 | 0.81 | 1110 | 0.73 | 1258 |
| Region 2 | 0.64 | 700 | 0.66 | 746 | 0.66 | 724 | 0.83 | 746 | 0.65 | 867 |
| Region 3 | 0.75 | 1093 | 0.78 | 1201 | 0.65 | 1157 | 0.91 | 1197 | 0.76 | 1346 |
| Region 4 | 0.81 | 1178 | 0.76 | 1185 | 0.84 | 1209 | 0.91 | 1346 | 0.72 | 1575 |
| Region 5 | 0.79 | 1476 | 0.8 | 1580 | 0.67 | 1556 | 0.88 | 1611 | 0.85 | 1876 |
| Region 6 | 0.73 | 729 | 0.73 | 750 | 0.89 | 724 | 0.82 | 851 | 0.39 | 948 |
| Region 7 | 0.73 | 415 | 0.77 | 441 | 0.64 | 449 | 0.7 | 479 | 0.38 | 503 |
| Region 8 | 0.73 | 510 | 0.66 | 604 | 0.76 | 577 | 0.87 | 521 | 0.66 | 574 |
| Region 9 | 0.77 | 1798 | 0.63 | 1752 | 0.8 | 1912 | 0.92 | 1947 | 0.87 | 2302 |
| Region 10 | 0.76 | 819 | 0.69 | 931 | 0.83 | 908 | 0.79 | 842 | 0.84 | 910 |
| Alaska | 0.13 | 27 | 0.24 | 32 | 0.29 | 33 | -0.01 | 28 | 0.12 | 27 |
| Alabama | 0.58 | 66 | 0.75 | 64 | 0.42 | 77 | 0.75 | 76 | 0.1 | 87 |
| Arkansas | 0.53 | 51 | 0.54 | 64 | 0.6 | 54 | 0.61 | 54 | 0.04 | 60 |
| Arizona | 0.63 | 204 | 0.5 | 207 | 0.64 | 220 | 0.67 | 214 | 0.87 | 267 |
| California | 0.78 | 1503 | 0.68 | 1447 | 0.78 | 1601 | 0.89 | 1637 | 0.82 | 1919 |
| Colorado | 0.57 | 237 | 0.54 | 249 | 0.65 | 275 | 0.63 | 262 | 0.45 | 283 |
| Connecticut | 0.44 | 163 | 0.62 | 165 | 0.15 | 182 | 0.4 | 175 | 0.37 | 199 |
| Delaware | 0.19 | 26 | 0.42 | 25 | -0.29 | 23 | -0.09 | 31 | 0.31 | 38 |
| Florida | *-* | 384 | *-* | 392 | *-* | 395 | *-* | 433 | *-* | 496 |
| Georgia | 0.64 | 182 | 0.7 | 200 | 0.57 | 197 | 0.69 | 193 | 0.08 | 222 |
| Hawaii | 0.18 | 34 | -0.13 | 39 | 0.16 | 31 | 0.58 | 34 | 0.15 | 40 |
| Iowa | 0.62 | 160 | 0.8 | 156 | 0.35 | 173 | 0.09 | 181 | 0.53 | 190 |
| Idaho | 0.52 | 50 | 0.57 | 64 | 0.74 | 51 | 0.53 | 53 | 0.06 | 60 |
| Illinois | 0.69 | 333 | 0.71 | 367 | 0.53 | 349 | 0.75 | 355 | 0.57 | 411 |
| Indiana | 0.63 | 147 | 0.65 | 158 | 0.53 | 152 | 0.65 | 161 | 0.51 | 191 |
| Kansas | 0.45 | 90 | 0.49 | 83 | 0.61 | 92 | 0.47 | 115 | -0.03 | 116 |
| Kentucky | 0.48 | 84 | 0.59 | 100 | 0.66 | 83 | 0.3 | 88 | 0.64 | 114 |
| Louisiana | 0.51 | 70 | 0.42 | 48 | 0.53 | 53 | 0.69 | 109 | 0.01 | 114 |
| Massachusetts | 0.75 | 576 | 0.77 | 406 | 0.46 | 631 | 0.77 | 678 | 0.82 | 766 |
| Maryland | 0.65 | 279 | 0.78 | 308 | 0.27 | 307 | 0.8 | 293 | 0.16 | 328 |
| Maine | 0.31 | 64 | 0.29 | 60 | 0.15 | 70 | 0.46 | 72 | 0.5 | 84 |
| Michigan | 0.48 | 271 | 0.45 | 270 | -0.02 | 287 | 0.64 | 296 | 0.77 | 381 |
| Minnesota | 0.65 | 163 | 0.83 | 182 | 0.24 | 163 | 0.7 | 190 | 0.71 | 216 |
| Missouri | 0.57 | 131 | 0.66 | 153 | 0.47 | 151 | 0.58 | 142 | 0.28 | 159 |
| Mississippi | 0.34 | 26 | 0.29 | 31 | 0.24 | 25 | 0.44 | 32 | 0.28 | 35 |
| Montana | 0.38 | 34 | 0.61 | 41 | -0.02 | 40 | 0.59 | 33 | 0.21 | 36 |
| North Carolina | 0.68 | 237 | 0.74 | 202 | 0.53 | 222 | 0.76 | 296 | 0.54 | 349 |
| North Dakota | 0.38 | 60 | 0.33 | 89 | 0.06 | 67 | 0.39 | 49 | -0.02 | 53 |
| Nebraska | 0.36 | 35 | 0.71 | 49 | -0.15 | 33 | 0.23 | 41 | 0.04 | 38 |
| New Hampshire | 0.49 | 59 | 0.57 | 60 | -0.11 | 66 | 0.6 | 68 | 0.31 | 73 |
| New Jersey | 0.57 | 196 | 0.56 | 226 | 0.43 | 203 | 0.75 | 205 | 0.65 | 253 |
| New Mexico | 0.6 | 96 | 0.7 | 77 | 0.7 | 88 | 0.52 | 102 | 0.36 | 156 |
| Nevada | 0.31 | 57 | -0.07 | 58 | 0.44 | 60 | 0.49 | 62 | 0.64 | 75 |
| New York | 0.62 | 442 | 0.71 | 478 | 0.57 | 456 | 0.78 | 481 | 0.51 | 551 |
| Ohio | 0.68 | 351 | 0.68 | 386 | 0.34 | 377 | 0.72 | 379 | 0.58 | 418 |
| Oklahoma | 0.55 | 125 | 0.72 | 157 | 0.48 | 138 | 0.49 | 133 | 0.09 | 127 |
| Oregon | 0.65 | 352 | 0.75 | 414 | 0.52 | 382 | 0.58 | 357 | 0.63 | 378 |
| Pennsylvania | 0.68 | 368 | 0.74 | 409 | 0.66 | 374 | 0.72 | 411 | 0.73 | 473 |
| Rhode Island | 0.4 | 58 | 0.59 | 54 | -0.02 | 60 | 0.17 | 71 | 0.47 | 80 |
| South Carolina | 0.46 | 88 | 0.26 | 88 | 0.48 | 93 | 0.65 | 110 | 0.29 | 111 |
| South Dakota | 0.43 | 23 | 0.51 | 21 | 0.22 | 28 | 0.79 | 26 | 0.16 | 26 |
| Tennessee | 0.45 | 112 | 0.25 | 108 | 0.36 | 117 | 0.74 | 119 | 0.26 | 161 |
| Texas | 0.68 | 388 | 0.73 | 404 | 0.85 | 392 | 0.72 | 453 | 0.24 | 491 |
| Utah | 0.67 | 131 | 0.69 | 178 | 0.55 | 143 | 0.58 | 124 | 0.61 | 137 |
| Virginia | 0.61 | 327 | 0.63 | 369 | 0.39 | 357 | 0.81 | 363 | 0.59 | 384 |
| Vermont | 0.36 | 38 | 0.58 | 33 | 0.34 | 38 | 0.51 | 46 | 0.49 | 56 |
| Washington | 0.61 | 390 | 0.47 | 421 | 0.64 | 441 | 0.71 | 403 | 0.75 | 446 |
| Wisconsin | 0.59 | 211 | 0.75 | 216 | 0.08 | 228 | 0.63 | 231 | 0.79 | 260 |
| West Virginia | 0.45 | 47 | 0.59 | 44 | 0.21 | 52 | 0.4 | 48 | 0.44 | 62 |
| Wyoming | 0.35 | 26 | 0.32 | 28 | 0.21 | 24 | 0.45 | 26 | 0.1 | 38 |
| Boston | 0.69 | 304 | 0.63 | 194 | 0.4 | 359 | 0.74 | 370 | 0.8 | 373 |

HHS regions: Region 1 Connecticut, Maine, Massachusetts, New Hampshire, Rhode Island, Vermont; Region 2: New Jersey, New York, Puerto Rico, US Virgin Islands; Region 3: Delaware, District of Columbia, Maryland, Pennsylvania, Virginia, West Virginia; Region 4: Alabama, Florida, Georgia, Kentucky, Mississippi, North Carolina, South Carolina, Tennessee; Region 5: Illinois, Indiana, Michigan, Minnesota, Ohio, Wisconsin; Region 6: Arkansas, Louisiana, New Mexico, Oklahoma, Texas; Region 7: Iowa, Kansas, Missouri, Nebraska; Region 8: Colorado, Montana, North Dakota, South Dakota, Utah, Wyoming; Region 9: Arizona, California, Guam, Hawaii, Nevada; and Region 10: Alaska, Idaho, Oregon, Washington

**Table S3.** Pearson correlations between CDC number positive viral reports and EHR, crowd-sourced (FNY), and CDC ILINet

| Geographical Resolution | All Seasons | | | 2012-13 | | | 2013-14 | | | 2014-15 | | | 2015-16 | | |
| --- | --- | --- | --- | --- | --- | --- | --- | --- | --- | --- | --- | --- | --- | --- | --- |
|  | EHR | FNY | ILINet | EHR | FNY | ILINet | EHR | FNY | ILINet | EHR | FNY | ILINet | EHR | FNY | ILINet |
| National | 0.90 | 0.70 | 0.89 | 0.97 | 0.70 | 0.96 | 0.97 | 0.92 | 0.97 | 0.98 | 0.96 | 0.98 | 0.99 | 0.92 | 0.88 |
| Region 1 | 0.85 | 0.66 | 0.83 | 0.92 | 0.76 | 0.94 | 0.9 | 0.84 | 0.9 | 0.96 | 0.89 | 0.97 | 0.85 | 0.66 | 0.83 |
| Region 2 | 0.94 | 0.59 | 0.85 | 0.87 | 0.65 | 0.95 | 0.9 | 0.74 | 0.86 | 0.97 | 0.9 | 0.95 | 0.94 | 0.59 | 0.85 |
| Region 3 | 0.94 | 0.73 | 0.93 | 0.95 | 0.71 | 0.98 | 0.95 | 0.8 | 0.96 | 0.98 | 0.94 | 0.99 | 0.94 | 0.73 | 0.93 |
| Region 4 | 0.85 | 0.67 | 0.91 | 0.94 | 0.46 | 0.95 | 0.96 | 0.71 | 0.95 | 0.91 | 0.86 | 0.97 | 0.85 | 0.67 | 0.91 |
| Region 5 | 0.82 | 0.60 | 0.77 | 0.89 | 0.69 | 0.93 | 0.93 | 0.92 | 0.91 | 0.98 | 0.88 | 0.98 | 0.82 | 0.60 | 0.77 |
| Region 6 | 0.90 | 0.68 | 0.87 | 0.97 | 0.63 | 0.95 | 0.88 | 0.82 | 0.96 | 0.92 | 0.84 | 0.95 | 0.90 | 0.68 | 0.87 |
| Region 7 | 0.89 | 0.64 | 0.84 | 0.8 | 0.57 | 0.81 | 0.73 | 0.72 | 0.91 | 0.93 | 0.82 | 0.59 | 0.89 | 0.64 | 0.84 |
| Region 8 | 0.90 | 0.68 | 0.87 | 0.95 | 0.71 | 0.98 | 0.92 | 0.83 | 0.93 | 0.89 | 0.8 | 0.83 | 0.90 | 0.68 | 0.87 |
| Region 9 | 0.93 | 0.63 | 0.80 | 0.95 | 0.58 | 0.94 | 0.94 | 0.93 | 0.93 | 0.96 | 0.93 | 0.94 | 0.93 | 0.63 | 0.80 |
| Region 10 | 0.85 | 0.66 | 0.88 | 0.68 | 0.63 | 0.93 | 0.88 | 0.76 | 0.94 | 0.82 | 0.78 | 0.94 | 0.85 | 0.66 | 0.88 |

HHS regions: Region 1 Connecticut, Maine, Massachusetts, New Hampshire, Rhode Island, Vermont; Region 2: New Jersey, New York, Puerto Rico, US Virgin Islands; Region 3: Delaware, District of Columbia, Maryland, Pennsylvania, Virginia, West Virginia; Region 4: Alabama, Florida, Georgia, Kentucky, Mississippi, North Carolina, South Carolina, Tennessee; Region 5: Illinois, Indiana, Michigan, Minnesota, Ohio, Wisconsin; Region 6: Arkansas, Louisiana, New Mexico, Oklahoma, Texas; Region 7: Iowa, Kansas, Missouri, Nebraska; Region 8: Colorado, Montana, North Dakota, South Dakota, Utah, Wyoming; Region 9: Arizona, California, Guam, Hawaii, Nevada; and Region 10: Alaska, Idaho, Oregon, Washington
